# A Novel Approach for the Treatment of Aerobic Vaginitis: Azithromycin Liposomes-in-Chitosan Hydrogel

**DOI:** 10.3390/pharmaceutics15051356

**Published:** 2023-04-28

**Authors:** Ana Čačić, Daniela Amidžić Klarić, Sabina Keser, Maja Radiković, Zora Rukavina, May Wenche Jøraholmen, Lidija Uzelac, Marijeta Kralj, Nataša Škalko-Basnet, Maja Šegvić Klarić, Željka Vanić

**Affiliations:** 1 Microbiology and Biology Laboratory, PLIVA Croatia Ltd., Prilaz Baruna Filipovića 25, 10000 Zagreb, Croatia; ana.cacic90@gmail.com; 2 Department of Pharmaceutical Technology, Faculty of Pharmacy and Biochemistry, University of Zagreb, A. Kovačića 1, 10000 Zagreb, Croatia; damidzic@pharma.hr (D.A.K.); skeser@pharma.hr (S.K.); mradikovic@gmail.com (M.R.); zpalac@pharma.hr (Z.R.); 3 Drug Transport and Delivery Research Group, Department of Pharmacy, Faculty of Health Sciences, University of Tromsø The Arctic University of Norway, Universitetsveien 57, 5037 Tromsø, Norway; may.w.joraholmen@uit.no (M.W.J.); natasa.skalko-basnet@uit.no (N.Š.-B.); 4 Department of Molecular Medicine, Ruđer Bošković Institute, Bijenička Cesta 54, 10000 Zagreb, Croatia; luzelac@irb.hr (L.U.); marijeta.kralj@irb.hr (M.K.); 5 Department of Microbiology, Faculty of Pharmacy and Biochemistry, University of Zagreb, A. Kovačića 1, 10000 Zagreb, Croatia; msegvic@pharma.hr

**Keywords:** azithromycin, liposomes, chitosan, hydrogel, aerobic vaginitis, vaginal drug delivery, antimicrobial activity, vaginal infections

## Abstract

Biocompatible mucoadhesive formulations that enable a sustained drug delivery at the site of action, while exhibiting inherent antimicrobial activity, are of great importance for improved local therapy of vaginal infections. The aim of this research was to prepare and evaluate the potential of the several types of azithromycin (AZM)-liposomes (180–250 nm) incorporated into chitosan hydrogel (AZM-liposomal hydrogels) for the treatment of aerobic vaginitis. AZM-liposomal hydrogels were characterized for in vitro release, and rheological, texture, and mucoadhesive properties under conditions simulating the vaginal site of application. The role of chitosan as a hydrogel-forming polymer with intrinsic antimicrobial properties was explored against several bacterial strains typical for aerobic vaginitis as well as its potential effect on the anti-staphylococcal activity of AZM-liposomes. Chitosan hydrogel prolonged the release of the liposomal drug and exhibited inherent antimicrobial activity. Additionally, it boosted the antibacterial effect of all tested AZM-liposomes. All AZM-liposomal hydrogels were biocompatible with the HeLa cells and demonstrated mechanical properties suitable for vaginal application, thus confirming their potential for enhanced local therapy of aerobic vaginitis.

## 1. Introduction

Aerobic vaginitis is a form of vaginitis accompanied by the growth of abnormal vaginal microflora comprising aerobic, enteric commensals, or pathogens (*Staphylococcus aureus*, *Streptococcus agalactiae*, *Escherichia coli*, *Pseudomonas aeruginosa*, and *Enterococcus faecalis*), in comparison to bacterial vaginosis, where mainly anaerobic microbes (*Gardnerella vaginalis*, *Prevotella species*, *Mycoplasma hominis*, and *Mobiluncus species*) are present [1,2]. Both types of vaginitis comprise a decrease in the dominance of lactobacilli and an increase in vaginal pH. However, aerobic vaginitis is accompanied by more extreme inflammatory changes and deficient epithelial maturation than bacterial vaginosis [2,3]. If untreated, aerobic vaginitis may lead to gynecological and obstetrical complications, including premature rupture of membranes, preterm delivery, fetal infections, infertility, abnormal Pap test results, and increased risk of sexually transmitted bacterial and viral infections [4,5].

Aerobic vaginitis is commonly treated with local and oral antibiotics, estrogens, non-steroidal anti-inflammatory drugs, and probiotics [2,6]. Local therapy is preferred as it enables higher drug concentration at the action site, while escaping systemic adverse effects and reducing the risk for bacterial resistance. Nevertheless, conventional formulations for vaginal administration are not always effective because of their leakage, messiness, inappropriate drug release, or short residence time inside the vagina. These limitations can be overcome by the use of nanoparticle-based mucoadhesive formulations [7,8,9]. Thus, liposomes encapsulating antimicrobials and incorporated into poly(acrylate) hydrogels have been shown to prolong retention of the formulation on vaginal mucosa, enabling sustained release of the liposomally-encapsulated drug [10]. Moreover, liposomes have been shown to improve delivery of the antibiotics to bacteria and biofilms as reported by decreased minimal inhibitory concentrations (MICs) and minimal biofilm inhibitory concentrations [11,12,13].

Hydrogels represent one of the most common vaginal dosage forms exerting a moisturizing effect, appropriate viscosity, increased residence time, and good distribution over the mucosal surface. They are well accepted by the patients and easy to produce [14,15]. Among the numerous hydrogel-forming polymers [16], chitosan is particularly interesting because of its mucoadhesiveness, biodegradability, and antimicrobial activity against various bacteria and fungi [17], which is of great importance considering the increased global trend of microbial resistance. The potentials of chitosan in enhanced vaginal drug delivery have been investigated in the form of various drug delivery nanosystems [17,18].

We previously demonstrated the suitability of liposomes with encapsulated azithromycin (AZM) for the treatment of bacterial cervicovaginal *Chlamydia trachomatis* and *E. coli* infections. All the tested liposomes were more effective against both planktonic and biofilm-forming *E. coli* than the free AZM [19]. Continuing this research line, we report here, for the first time, the potential of chitosan hydrogel (chitosan-HG) as a vehicle for AZM-liposomes intended for the local treatment of aerobic vaginitis. Specifically, incorporating AZM-liposomes into the chitosan-HG should increase their retention within the vaginal cavity, prolong drug release, and potentially improve the antimicrobial effect.

Chitosan-HG was assessed in vitro for its intrinsic antimicrobial potential against three bacterial strains (*S. aureus*, *E. coli*, *P. aeruginosa*), characteristic for aerobic vaginitis, and against yeast *Candida albicans*, whose overgrowth is associated with vulvovaginal vaginitis. Moreover, to determine whether the chitosan-HG could potentiate the antimicrobial activity of the several types of AZM-liposomes, in vitro antibacterial studies against *S. aureus* ATCC 6538 were performed, and the results were compared with the antibacterial activities of the different types of AZM-liposomes, chitosan-HG incorporating free AZM (control-HG), and the originally prepared chitosan-HG. The AZM-liposomes-in-chitosan-HG formulations (AZM-liposomal hydrogels) were further evaluated for in vitro drug release in conditions simulating the vaginal environment. In addition, the rheological, texture, and mucoadhesive properties of the AZM-liposomal hydrogels were determined to consider the applicability of the tested formulations for vaginal administration. Finally, the biocompatibility of the developed formulations was examined in vitro on the HeLa cells.

## 2. Materials and Methods

### 2.1. Materials

AZM in the form of dihydrate was a donation from PLIVA Croatia Ltd. (Zagreb, Croatia). Egg phosphatidylcholine (EPC), egg phosphatidylglycerol sodium salt (EPG), hydrogenated soybean phosphatidylcholine (SPC-3), and monoacyl phosphatidylcholine from soybean (SLPC-80) were kindly gifted by Lipoid GmbH (Ludwigshafen, Germany). Medium molecular weight (MMW) chitosan with 82.0% deacetylation degree and viscosity of 420 CPS (c = 1%, 1% acetic acid) as well as Sephadex G-50, urea, glucose, lactic acid, propylene glycol, Dulbecco’s modified Eagle medium, fetal bovine serum, L-glutamine, and bovine serum albumin were obtained from Sigma-Aldrich (St. Louis, MO, USA). Acetonitrile, ethanol, and methanol were of HPLC grade and procured from BDH Prolabo (Lutterworth, UK). Tryptic Soy Agar, Sabouraud Dextrose Agar and Tryptic Soy Broth were purchased from BioMérieux (Marcy-l’Étoile, France). Dey/Engley (D/E) Neutralizing Broth and Difco Antibiotic Medium 11 were obtained from Becton Dickinson (East Rutherford, NJ, USA).

Phosphate buffer, pH 8.0 (used in microbiological studies), was prepared by dissolving 16.73 g of potassium hydrogen phosphate and 0.523 g of potassium dihydrogen phosphate in demineralized water up to 1000 mL.

Vaginal fluid simulant (VFS, pH 4.5) and phosphate buffer (pH 7.5) were prepared as previously reported [19].

### 2.2. Preparation of Chitosan-HG

Chitosan (4 g) was weighted in a beaker and dispersed in 44 g of 3.5% (*w*/*w*) lactic acid. Subsequently, propylene glycol (10 g) was added, and the mixture was manually stirred, followed by the addition of the appropriate amount of demineralized water to attain the final concentration of the chitosan 4% (*w*/*w*). The blend was continued to stir, and bath sonicated until a homogenous mixture was formed (approx. 45 min). The hydrogel was covered with parafilm and allowed to swell for 24 h at room temperature.

### 2.3. Preparation and Characterization of AZM-Liposomes

Three preparations of AZM-liposomes differing in composition (Table 1) and bilayer properties, namely, conventional liposomes (CLs) characterized by rigid bilayers and two preparations of elastic liposomes, i.e., propylene glycol liposomes (PGLs) and deformable propylene glycol liposomes (DPGLs), were prepared according to procedures described in detail by Vanić et al. [19]. All the liposomes were manually extruded (3 cycles) through 400 nm pore-sized polycarbonate membranes using LiposoFast Basic extruder (Avestin, Ottawa, ON, Canada), and stored at 4 °C prior to their use.

Mean diameters, polydispersity indexes, and zeta potentials of the AZM-liposomes were analyzed by dynamic light scattering at 25 °C on a previously calibrated Zetasizer 3000HS (Malvern Instruments, Malvern, UK). The measurements were performed using a capillary cell with an optical modulator at 1000 Hz (zeta potential) and scattering angle of 90° (size analysis). Samples of the liposomes were adequately diluted with buffer (pH 7.5) to ensure a suitable count rate.

To determine the elasticity of the liposomal bilayers, continuous extrusion of the liposomal dispersions through 100 nm pore-sized membranes was performed at constant pressure (2.5 bar) for 5 min, and the degree of the bilayer elasticity was calculated according to the previously reported formula [19].

Quantification of the liposomally-encapsulated AZM was performed by HPLC at 210 nm (C18 column; acetonitrile/phosphate buffer, pH 7.5 in ratio 70:30 *v*/*v* as mobile phase) [13] after separation of the nonencapsulated drug by the minicolumn centrifugation method [19]. Encapsulation of the drug in liposomes was expressed as µg of encapsulated AZM per mg of the phospholipids used.

### 2.4. Preparation of AZM-Liposomal Hydrogels

Each type of AZM-liposomes (CLs, PGLs, or DPGLs) was incorporated into the chitosan-HG by manual stirring for approximately 5 min (room temperature) until formation of a homogenous AZM-liposomal hydrogel (CLs-HG, PGLs-HG, or DPGLs-HG). The concentration of the AZM-liposomes inside the hydrogel was 30% *w*/*w* (AZM-liposomes/AZM-liposomal hydrogel).

Control-HG was prepared under the same conditions and contained a solution of the free AZM in the same concentration as in the liposomes. AZM-solution was prepared by dissolving the drug in a mixture of ethanol and water (6/4, *v*/*v*) and subsequent dilution with buffer, pH 7.5 to achieve appropriate concentration of the AZM-solution inside the hydrogel (30%, *w*/*w*). Control-HG was examined in all the studies where AZM-liposomal hydrogels were explored.

### 2.5. Measurements of the Hydrogels’ pH Values

The pH values of all the prepared hydrogels were determined at 25 °C by a pH meter equipped with an electrode for semisolid formulations (Mettler-Toledo, Greifensee, Switzerland). The pH values were determined by direct immersion of the pH electrode for semisolid formulations inside the hydrogels. Prior to the measurement, the pH meter was calibrated at 25 °C using standard buffer solutions (pH 4.0, pH 7.0, and pH 9.0). Three measurements were performed for each sample of the hydrogel.

### 2.6. In Vitro Release Studies

A sample of the AZM-liposomal hydrogel (CLs-HG, PGLs-HG or DPGLs-HG) or control-HG, each corresponding to 0.5 mg AZM, was placed into a dialysis bag (Medicell Membranes, Mw cut-off 3500 Da) and dialyzed against 20 mL of VFS during constant stirring (150 rpm) at 37 °C. At certain time intervals (1, 2, 3, 4, 5, 6, and 24 h), 2 mL of the dialysis medium containing the released drug was withdrawn, filtered through 0.22 µm Minisart RC4 filters (Sartorius AG, Göttingen, Germany), and replaced with the fresh medium (VFS). The amount of the released AZM was determined by HPLC as described earlier [19].

### 2.7. Rheological Assessment of the AZM-Liposomal Hydrogels

The rheological characteristics of the AZM-liposomal hydrogels were determined at 25 and 37 °C using a Modular Compact Rheometer MCR 102 (Anton Paar GmbH, Graz, Austria) fitted with a parallel-plate measuring system (diameter 25 mm, PP25), and the gap set to 1 mm. Prior to the measurements, the AZM-liposomal hydrogels were equilibrated for 10 min at the corresponding temperature. Viscosity curves were determined by rotational tests performed in the shear rate range from 0.1 to 1000 s^−1^. To simulate in vivo vaginal application, the AZM-liposomal hydrogels were mixed with VFS in 5:1 ratio (*w*/*w*), as previously reported [10], and the measurements were performed at 37 °C. Oscillatory amplitude sweep tests were also carried out with the hydrogels mixed with VFS (5:1, *w*/*w*) at 37 °C applying an angular frequency of 10 s^−1^ in the shear strain range of 0.1–100%. Control-HG was examined under the same conditions.

### 2.8. Texture Analysis of the AZM-Liposomal Hydrogels

Texture properties of the AZM-liposomal hydrogels were assessed by a TA.XT Plus Texture Analyser (Stable Micro Systems Ltd., Surrey, UK) using a previously reported procedure [10]. In brief, 50 g of the hydrogel was placed in a standard beaker, ensuring that no air bubbles were included and that the surface was smooth. A disk (40 mm in diameter) was pushed into the formulation (10 mm at a speed of 1 mm/s) and removed. The hardness and cohesiveness of each AZM-liposomal hydrogel were determined. The results were compared with the texture characteristics of the control-HG and chitosan-HG. Each formulation was measured five times, ensuring the same conditions for each measurement.

### 2.9. Mucoadhesion Test

The mucoadhesive properties of the different AZM-hydrogels (CLs-HG, PGLs-HG, DPGLs-HG, and control-HG) were evaluated using the TA.XT Plus Texture Analyser equipped with the gel mucoadhesion probe (Stable Micro Systems Ltd., Surrey, UK). The experiments were performed on porcine vaginal mucosa, which was obtained as waste in a local slaughterhouse. The vaginal mucosa was carefully separated from the underlying tissue, washed with isotonic solution (0.9% NaCl), cut into smaller pieces, and frozen at −20 °C. Immediately prior to the measurements the mucosa was defrosted at room temperature, cut into squares of approximately 6.25 cm^2^, and soaked in VFS for 5 min. Each mucosal sample was attached to the lower platform and incubated at 37 °C. The tested hydrogel (1.5 mL) was attached to the gel mucoadhesion probe, facing the vaginal mucosa samples. The probe contained a conically cut area (15 mm diameter) with concentric grooves, which promoted gel attachment.

AZM-hydrogel was placed in contact with the vaginal mucosa for 30 s using a force of 0.05 N. The probe was lowered at a speed of 0.5 mm/s until it reached the sample surface. Test speed and probe withdrawing speed were both set at 0.1 mm/s. A trigger force of 0.029 N and return distance of 15 mm were applied.

The Texture Analyser software (Exponent Connect Version 7.0.6.0) was used to calculate the maximum detachment force and work of adhesion (the area under the force/distance curve), which served as an indicator of the mucoadhesive properties.

All measurements were performed in triplicate.

### 2.10. Antimicrobial Evaluation of the Chitosan-HG

Intrinsic antimicrobial activity of the chitosan-HG was evaluated toward *S. aureus* ATCC 6538, *P. aeruginosa* ATCC 9027, *E. coli* ATCC 8739, and *C. albicans* ATCC 10231. The assessment was performed based on the logarithmic reduction of the number of test microbes relative to their initial number using the pharmacopoeial method for testing the effectiveness of preservatives in preparations for topical administration [20].

Briefly, samples of chitosan-HGs (20 g each) were inoculated with 0.1 mL of bacterial or 0.2 mL yeast suspension. The final concentration of test microbes in each hydrogel sample was 10^5^–10^6^ colony-forming units/g (CFU/g) microbes.

The samples were well mixed to achieve a homogeneous distribution of the microbes within the hydrogels and were stored at 20–25 °C, protected from light, for 28 days. The number of test microbes in the inoculated chitosan-HG sample was determined immediately after inoculation and at the particular time intervals (7th, 14th, and 28th days), according to the pharmacopoeial pour-plate method [20]. For that purpose, 1 g of the inoculated hydrogel sample was weighed for each test microorganism and serial dilutions of 10^−1^ to 10^−5^ were prepared in tubes containing 9 mL of D/E neutralizing broth. The number of microbes was determined by the pour-plate method whereby 1 mL of each dilution of the test sample was placed in a Petri dish in duplicate and homogenized with 15–20 mL of the previously dissolved growth medium. Tryptic soy agar and Sabouraud’s dextrose agar were used for bacterial and yeast growth, respectively. Incubation was performed for 3–5 days at 30–35 °C (bacteria) or 5 days at 20–25 °C for fungi.

During the antimicrobial activity assessment of the chitosan-HG, in parallel, negative and positive control tests were performed, too. Negative control represented the microbiological examination of the chitosan-HG not inoculated with the microbes, while the inoculum control of tested microbes served as positive control.

After incubation of the chitosan-HGs with the test microbes, grown colonies were counted and the number of the test microbes in 1 g of the tested hydrogel sample was calculated according to the following equation:

$$X\;\;\;\frac{1}{10^{-n}},$$

where X represents the number of colonies grown in each dilution, and n denotes the dilution of the test microorganism.

Based on the number of microbes determined, the corresponding logarithmic (log_10_) value was calculated in 1 g of the tested hydrogel sample.

### 2.11. Antibacterial Activity of the AZM-Liposomal Hydrogels

In vitro antibacterial activities of the different AZM-liposomal hydrogels (CLs-HG, PGLs-HG, and DPGLs-HG) toward *S. aureus* ATCC 6538 were determined by the agar-diffusion method. To determine whether chitosan-HG could potentiate the antibacterial activity of the incorporated AZM-liposomes, the studies were also performed with AZM-liposomes (CLs, PGLs, and DPGLs), a solution of AZM in an ethanol/water mixture (6/4, *v*/*v*), control-HG (hydrogel containing AZM-solution instead of the AZM-liposomes in the same concentration as in the liposomes), chitosan-HG, and an ethanol/water solution (6/4, *v*/*v*) that represented the solvent control. To enable diffusion of the hydrogel into agar, all the hydrogel samples were diluted with buffer, pH 8.0.

The following procedure was applied: The inoculum of the tested bacteria in Tryptic soy broth at a concentration of 1 × 10^8^ CFU/mL was diluted with a 0.9% saline solution (1:10) and then evenly dispersed in liquid Antibiotic medium 11 (1:100) thermostated at 37 °C. Inoculated agar (20 mL) was poured into a sterile Petri plate (100 × 15 mm) and left on a flat surface to solidify. Subsequently, six wells (each 6 mm in diameter) were drilled on each plate using sterilized stainless steel tubes and approximately 0.1 mL of the diluted tested sample (corresponding to 1.2 µg AZM) was added into the wells. The plates were incubated at 37 °C under aerobic conditions for 24 h. Following incubation, confluent growth of the bacteria on the plates was observed, and the diameters of the bacterial growth inhibition zones were measured in millimeters. If the zone of inhibition was not present around the well, the tested material was considered to have no antibacterial effect on the tested bacterial strain.

These experiments were conducted in duplicate.

### 2.12. In Vitro Biocompatibility Assessment

The HeLa cell line was obtained from the American Type Culture Collection (CCL-2™). HeLa cells were cultured as monolayers and maintained in Dulbecco’s modified Eagle’s medium supplemented with 10% fetal bovine serum, 2 mM L-glutamine, 100 U/mL penicillin, and 100 μg/mL streptomycin in a humidified atmosphere with 5% CO_2_ at 37 °C.

The cells were inoculated onto a series of standard 96-well microtiter plates at concentrations of 1.6 × 10^4^ cells/mL. The following day, prediluted AZM-liposomal hydrogels or control-HG were added at five concentrations (1, 5, 10, 20, and 40 μg/mL) and incubated with the cells for an additional 24 h (37 °C, 5% CO_2_). The corresponding empty liposomal hydrogels (without AZM) and empty control-HG (without AZM) were tested under the same conditions. The cell growth rate was evaluated by detecting the dehydrogenase activity in viable cells using the MTT assay, as previously reported [19]. The cell viability of the treated cells was displayed as a percentage compared to the untreated control cells. Each result represents a mean value from at least three separate experiments performed in quadruplicate.

### 2.13. Statistical Analysis

Statistical data analysis was evaluated by one-way ANOVA and Tukey’s multiple comparison test using the GraphPad 5.0 Prism program (GraphPad Software, Inc., San Diego, CA, USA). Means were considered significantly different when *p* < 0.05.

## 3. Results and Discussion

When designing a vaginal drug formulation, several factors affecting the success of therapy should be considered, ranging from precise diagnosis, selection of an effective drug in the appropriate dose, and finally, a formulation that remains long enough on the vaginal tissue, ensuring adequate release of the drug [7,19]. Topical formulations made from biodegradable and biocompatible excipients that additionally exhibit intrinsic antimicrobial activity are particularly valuable for the treatment of aerobic vaginitis. Among them, chitosan has shown great potential because of its natural origin, ability to form hydrogel, acidity suitable for the vaginal mucosa, and diversity of biological activities including anti-inflammatory and antimicrobial properties [21].

AZM-liposomes have already shown promise for treating cervicovaginal infections [19]. However, their liquid nature and non-mucoadhesive features limit vaginal application despite their therapeutic potential. Since chitosan-HGs have shown compatibility with liposomes intended for skin therapy [22], it seemed reasonable to use chitosan-HG as a vehicle (base) for incorporating AZM-liposomes intended for the local treatment of aerobic vaginitis.

The optimized AZM-liposomes [19], i.e., CLs, PGLs, and DPGLs, were incorporated into chitosan-HG, resulting in 30% AZM liposomes-in-hydrogel (*w*/*w*). As summarized in Table 2, all liposomes were anionic (zeta potential of approximately −60 mV), allowing good physical stability with mean diameters in the range of 180–250 nm and encapsulation efficacy between 6 and 8 µg AZM/mg lipid. CLs were the biggest and characterized by rigid bilayers in comparison to PGLs and DPGLs, which had elastic bilayers. Moreover, the presence of propylene glycol in PGLs and DPGLs led to the slightly increased encapsulation of AZM in comparison to CLs, probably because of its solubilizing effect.

### 3.1. Characterization of AZM-Liposomal Hydrogels

The physiological and anatomical features of the vagina should be respected when designing a vaginal formulation [7]. Since the healthy vaginal pH is slightly acidic [23] and increases during infections [2], the application of a formulation exerting a suitable pH value can be beneficial for local therapy of aerobic vaginitis.

**Figure 1:**
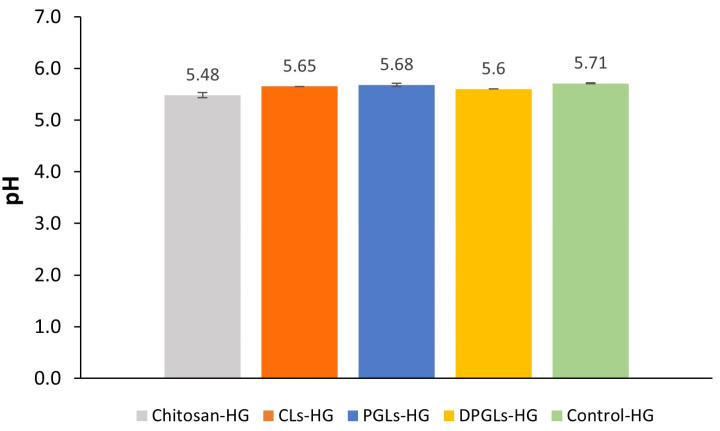
pH values of the originally prepared chitosan-HG and different AZM-hydrogels. The results represent the mean ± S.D. (*n* = 3). CLs-HG, conventional AZM-liposomes-in-chitosan-HG; control-HG, AZM-solution-in-chitosan-HG; DPGLs-HG, deformable propylene glycol AZM-liposomes-in-chitosan-HG; PGLs-HG, propylene glycol AZM-liposomes-in-chitosan-HG.

The chitosan-HG evaluated in this research was composed of 4% (*w*/*w*) MMW chitosan and contained lactic acid, a natural compound found in vaginal fluid [24]. Lactic acid was used for dissolving the chitosan during hydrogel preparation, instead of the commonly used acetic acid [25]. The hydrogel pH value was slightly acidic (5.48) and favorable for intended administration as well as incorporation of liposomes. As shown in Figure 1, the incorporation of AZM-liposomes resulted in slightly increased pH values because of the effect of the buffer pH 7.5 in which the liposomes were prepared; however, this change was not statistically significant (*p* > 0.05).

Volume, pH, and the constituents of vaginal fluid may affect drug release characteristics as well as the distribution and retention of the hydrogel within the vagina. Once the AZM-liposomal hydrogel is administered, it should distribute along the mucosa and provide prolonged residence time to enable delivery of the incorporated drug [7,10]. Therefore, performing in vitro release studies in conditions that simulate vaginal application, rheological and texture evaluation, including mucoadhesion testing, seem necessary in the characterization of the prepared AZM-hydrogels.

**Figure 2:**
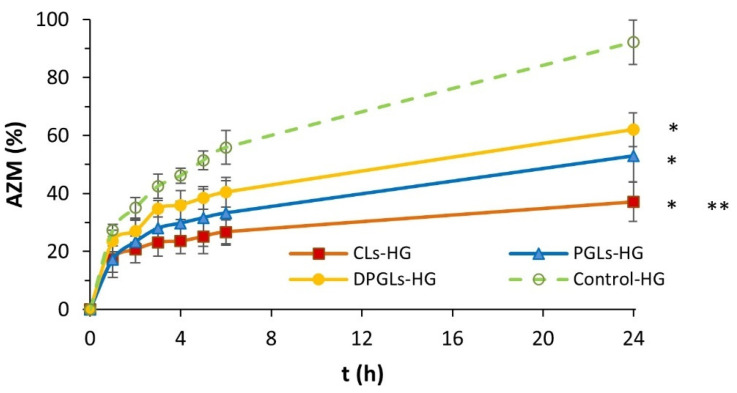
Cumulative in vitro release of AZM from the different AZM-hydrogels. The values represent the mean ± S.D. (*n* = 3). * Significantly different compared to control-HG at the 24 h time point (*p* < 0.05). ** Significantly different from DPGLs-HG at the 24 h time point (*p* < 0.05).

The dialysis tubing method was applied to study the release of AZM from the different AZM-liposomal hydrogels (CLs-HG, PGLs-HG, DPGLs-HG). The results displayed in Figure 2 demonstrated the prolonged release of AZM from all the liposomal hydrogels in comparison to control-HG (*p* < 0.05). Among the different types of liposomes incorporated into hydrogel, the slowest release of AZM was achieved by CLs-HG (*p* < 0.05), while somewhat faster release was obtained from PGLs-HG and DPGLs-HG. These results are associated with the bilayer properties of AZM-liposomes. Hence, with the increasing bilayer elasticity of AZM-liposomes (Table 2), a higher amount of AZM was released (Figure 2). The similar trend of AZM release was obtained from the AZM-liposomal suspensions (CLs, PGLs, and DPGLs); however, the ratio of the released drug was significantly greater [19], compared to the AZM-liposomal hydrogels because of the presence of a hydrogel barrier further extending release of the liposomal drug.

Assessment of rheological and texture features plays an important role in development of semisolid vaginal formulation. Specifically, rheological behavior affects the formulation appearance, its performance, stability, spreadability, and retention at the application site [26]. Moreover, understanding the rheological features of a formulation helps in explaining the relationship between its chemical structure and the resulting formulation properties [27]. For instance, viscosity measurements allow determination of the gel’s resistance to structural breakdown [26].

Vaginal hydrogels are expected to maintain appropriate viscosity upon application of higher shear rates or when diluted with vaginal fluid. Interaction of the formulation with vaginal fluid as well as body temperature can change the hydrogel’s initial viscosity [10]. Hence, viscosity measurements of the different AZM-hydrogels were performed at 25 °C and 37 °C, referring to storage and application temperatures, respectively.

**Figure 3:**
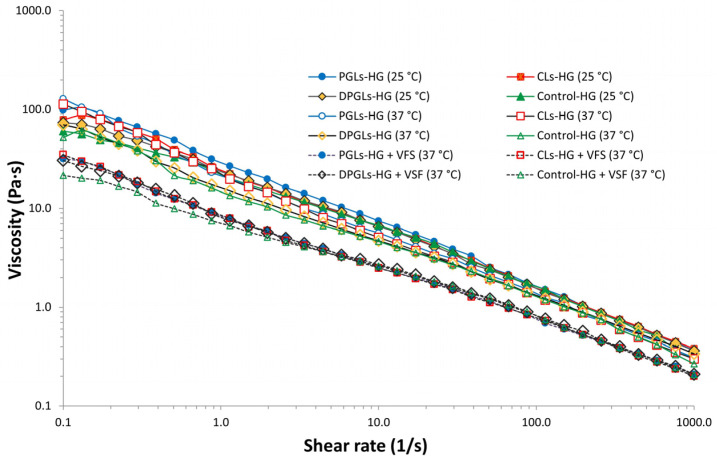
Viscosity profiles of different AZM-liposomal hydrogels at 25 °C and mimicked vaginal conditions (+VFS, 37 °C).

As can be seen in Figure 3, all tested AZM-hydrogels were of similar viscosities and exhibited shear-thinning profiles like those obtained with Carbopol gels [28]. The type of AZM-liposomes incorporated in chitosan-HG did not cause significant differences in the viscosity profiles of the different AZM-liposomal hydrogels. Similar behavior was also demonstrated with Carbopol hydrogels containing metronidazole- or clotrimazole-liposomes [10]. The slightly higher initial viscosities of the AZM-liposomal hydrogels compared to control-HG (Figure 3) were probably caused by the liposome membrane ingredients known to increase the strength of the hydrogel into which they are mixed [29].

To better simulate the in vivo performance of the AZM-hydrogels, VFS was added to all tested samples and viscosity profiles were determined at 37 °C. As a result, a significant decrease in the initial viscosities of the hydrogels (at 25 °C) was obtained (Figure 3). However, this reduced viscosity of the AZM-liposomal hydrogels was still considered suitable for retaining the formulation on vaginal mucosa. Our findings were in agreement with the previous research where the viscosity of liposomes incorporated in Carbopol gel was also assessed in simulated vaginal conditions [10] and are speculated to be the result of only dilution of the AZM-hydrogels with VFS.

Oscillatory tests are preferred in rheological evaluation as they provide information on the viscoelastic character of the semisolid formulation [26,27]. Like viscosity assessment, amplitude sweep measurements of the different AZM-liposomal hydrogels were also conducted in a mimicked vaginal environment [10].

**Figure 4:**
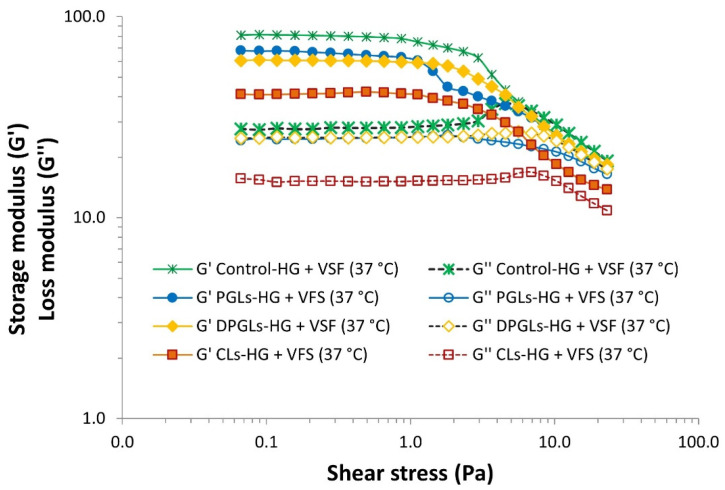
Amplitude sweep curves of the different AZM-liposomal hydrogels under mimicked vaginal conditions (+VFS, 37 °C).

AZM-hydrogels exhibited a linear viscoelastic region, i.e., a constant plateau where storage modulus (G’) and loss modulus (G″) values do not depend on the strain and only correlate with a molecular structure [26]. As demonstrated in Figure 4, all examined hydrogels demonstrated the higher storage modulus (G’), thus proving their viscoelastic solid structure. Among the different formulations, PGLs-HG, DPGLs-HG, and control-HG exhibited similar viscoelastic behavior, while CLs-HG displayed lower values (Figure 4), indicating its stronger structure. Specifically, CLs are composed of hydrogenated phospholipids (Table 1) contributing to the rigidity of their bilayers in comparison to PGLs and DPGLs, characterized by elastic membranes (Table 2). The results were supported by the findings of Mourtas et al. [29], where the elastic character of the gel strengthened with an increase in the concentration of hydrogenated phospholipids.

**Figure 5:**
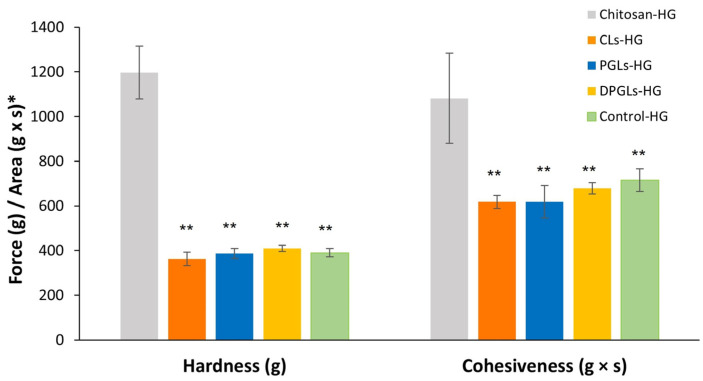
Texture properties of the different hydrogels, expressed as hardness and cohesiveness. * Force (g) refers to the hardness, while Area (g × s) refers to the cohesiveness. The presented values are the mean ± S.D. (*n* = 5). ** Significantly different compared to the chitosan-HG (*p* < 0.05).

The mechanical properties of the prepared AZM-liposomal hydrogels were also assessed by texture analysis. For this purpose, the hardness, corresponding to the easiness of administration at the application site [10], was determined as well as the cohesiveness, which refers to the recovery of the structural network within the hydrogel after application [22]. These properties can be affected by the ingredients of the formulation. In this study, the influence of incorporating different types of AZM-liposomes on the textural properties of chitosan-HG was examined, and the results were compared with the textural properties of the hydrogel containing the AZM-solution (control-HG). As expected, incorporation of 30% (*w*/*w*) AZM-liposomes into the chitosan-HG resulted in a significant decrease in the initial hardness and cohesiveness of the chitosan-HG (Figure 5). This effect was mostly caused by simple dilution of the chitosan-HG because a similar effect was observed with control-HG (which contained 30% AZM-solution, *w*/*w*). Comparison of the different AZM-liposomal hydrogels showed very similar profiles of hardness and cohesiveness regardless of the type of liposomes embedded in the hydrogel, while the cohesiveness of DPGLs-HG was negligibly higher than that obtained for CLs-HG and PGLs-HG (*p* > 0.05).

**Figure 6:**
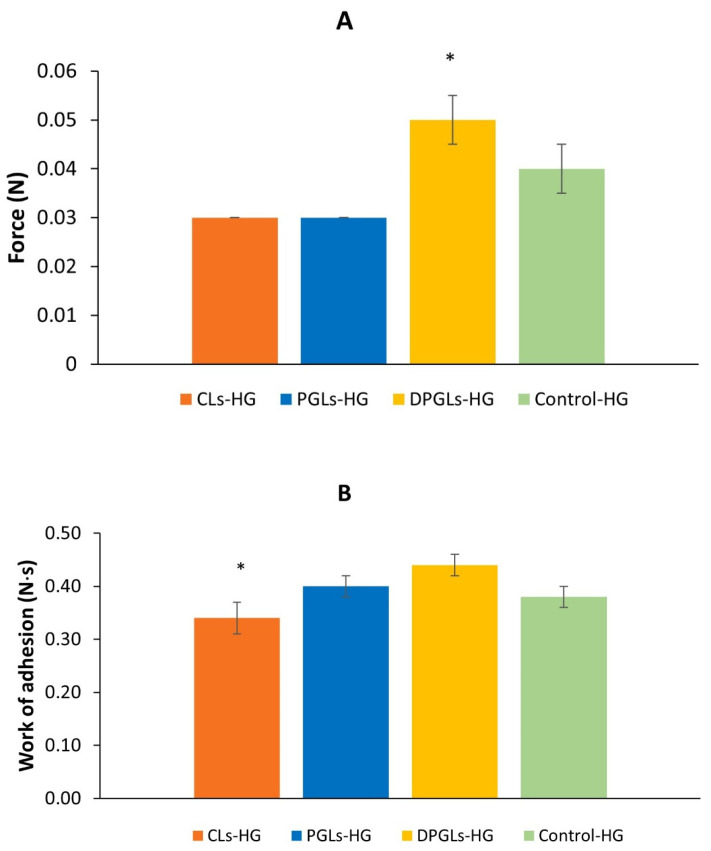
Mucoadhesion of different AZM-hydrogels on porcine vaginal mucosa: force detachment values (**A**) and work of adhesion (**B**). The values are the mean ± S.D. (*n* = 3). * Significantly different (*p* < 0.05).

All AZM-liposomal hydrogels showed prolonged release of AZM at simulated vaginal conditions (Figure 2). However, to permit prolonged contact of the liposomal drug at the vaginal mucosa, as a prerequisite for therapeutic effect, AZM-liposomal hydrogels should be sufficiently mucoadhesive. Mucoadhesion was estimated by a tensile test using a texture analyzer [30], where the measurement of maximum force, i.e., detachment force (Figure 6A), or the work required for detachment of the hydrogel sample from porcine vaginal mucosa, i.e., work of adhesion (Figure 6B), was determined.

The detachment force was found to be the highest for DPGLs-HG (*p* < 0.05), followed by control-HG, while the lowest values were determined for CLs-HG and PGLs-HG (Figure 6A). Therefore, it seems that the presence of only 15% lysophospholipids (SLPC-80) in bilayers of DPGLs had an impact on the mucoadhesivity of DPGLs-HG. We suppose that its contribution to mucoadhesivity is probably coupled to the role of lysophospholipids in the adhesion processes in biological tissues [31], but it should be further evaluated. The dominance of DPGLs-HG was additionally confirmed when the work of adhesion was measured (Figure 6B). However, it appeared that propylene glycol also contributed to mucoadhesivity because both PGLs and DPGLs contained the same portion of propylene glycol in comparison to CLs, which were composed of hydrogenated phospholipids and prepared without propylene glycol.

### 3.2. Antimicrobial Potential of the Chitosan-HG

The assessment of the intrinsic antimicrobial activity of the chitosan-HG was performed using the pharmacopeial method for determination of the effectiveness of preservatives in non-sterile formulations for topical and mucosal administration [20]. The study included all the requested bacterial strains (*S. aureus* ATCC 6538, *P. aeruginosa* ATCC 9027, *E. coli* ATCC 8739) and yeast (*C. albicans* ATCC 10231), while the efficacy against the mold (*Aspergillus brasiliensis* ATCC 1604) was not tested, as it was not of interest as a vaginal pathogenic microbe. The tested microbes are characteristic for aerobic vaginitis (*S. aureus*, *E. coli* and *P. aeruginosa*) [2] and vulvovaginal candidiasis (*C. albicans*) [7].

As shown in Table 3, chitosan-HG exhibited a similar intrinsic antimicrobial potential regardless of whether it was Gram-positive or Gram-negative bacteria or yeasts. Logarithm reduction of the bacterial and yeast number upon incubation in chitosan-HG met the criteria of the pharmacopoeia for preservatives in topical and mucosal formulations; the number of microbes dropped from the initial inoculum for about 4.5 logs on the 2nd, 7th, and 14th days, with no change between the 14th and 28th days of incubation.

The antimicrobial activity of chitosan has been linked to several mechanisms. The polycationic structure of chitosan in an acidic environment is thought to act through binding to the negatively charged bacterial cell wall components such as teichoic acid, lipopolysaccharide, and protein structures, leading to disruption of the cell wall. Furthermore, chitosan can attach to DNA and inhibit DNA replication, subsequently causing cell death [32,33]. Chitosan may also act as a chelating agent of trace metal elements causing toxin production and inhibition of microbial growth [34]. Chitosan inhibits the growth of *Candida* spp. by interfering in the action of synthase complexes responsible for the synthesis of key cell wall components (glucans and chitin). Antifungal activity in yeasts is more pronounced in limited carbon and nitrogen environments (e.g., in blood). Additionally, induction of the intracellular production of reactive oxygen species (ROS) and growth inhibition were seen under glucose starvation in *Candida glabrata*, linking limited nutrient content and ROS with the antifungal action of chitosan (reviewed in [35]).

Considering the microbial count reduction upon incubation in chitosan-HG and its slightly acidic pH (Figure 1) promoting the polycationic state of chitosan, such hydrogels can be applied as a vehicle (base) in the design of vaginal formulations without additional preservatives in the formulation and can favorably affect the vaginal mucosa because of its hydrophilic nature.

Interestingly, besides the antibacterial effect against bacteria typical for aerobic vaginitis (*E. coli*, *S. aureus*, *P. aeruginosa*), chitosan-HG exerted anticandidal activity, too. (Table 3). These effects of the chitosan-HG can be useful in the regulation of vaginal microbiota disorders and during antibacterial therapy when *C. albicans* overgrowth may occur, thus preventing its incidence.

### 3.3. Antimicrobial Activity of the AZM-Liposomal Hydrogels against S. aureus

The antimicrobial activity of AZM-liposomes and AZM-liposomal hydrogels was tested on *S. aureus* because this species is frequently involved in aerobic vaginitis [1,2]. Results of the agar-diffusion test (Table 4) indicated that all AZM-liposomes exhibited stronger antibacterial activity than the free drug. Comparison of the different types of AZM-liposomes demonstrated that CLs produced slightly larger inhibition zones of bacterial growth in comparison to DPGLs and/or PGLs and particularly when compared to control (AZM-solution). These findings are probably a result of the (phospho)lipid composition and the presence of propylene glycol affecting the bilayer properties of AZM-liposomes. The empty liposomes and empty liposomal hydrogels (both without AZM), which were used as controls in this study, failed to show any anti-staphylococcal effect. This observation was in accordance with our earlier report on the activity of AZM incorporated in anionic and cationic liposomes against *S. aureus* and methicillin-resistant *S. aureus* (MRSA) clinical isolates aimed at the treatment of skin infections [13], where the activity of the drug-free liposomes did not show a direct antimicrobial effect. The same research group also demonstrated that the proportion of propylene glycol in PGLs with AZM contributed to their antibacterial potential [13].

The AZM-liposomes used in this study, i.e., CLs, PGLs, and DPGLs, were previously examined against several strains of planktonic and biofilm-forming *E. coli* [19]. All types of AZM-liposomes were similarly effective against planktonic bacteria, but their activity differed against biofilm-forming *E. coli*. Thus, CLs were more effective at preventing formation of *E. coli* biofilms, while DPGLs were superior in their eradication. The greater activity of DPGLs against already formed biofilms is assumed to be a result of the presence of monoacyl phospholipids (lysophospholipids), which have been shown to play a role in adhesion processes in biological environment [31] and therefore could affect adhesion of DPGLs to biofilms. This effect was supported by this study where DPGLs-HG were shown to be the most mucoadhesive among the tested AZM-hydrogels (Figure 6).

Incorporation of AZM-liposomes into chitosan-HG improved the anti-staphylococcal activity of the corresponding AZM-liposomes. Among the different AZM-liposomal hydrogels, CLs-HG exerted the most potent activity against *S. aureus*, followed by PGLs-HG and DPGLs-HG. The observed increase in the antibacterial activity of AZM-liposomes by their incorporation into chitosan-HG can be linked to the chitosan polycationic structure discussed above. These findings were also supported by Hemmingsen et al. [22], who demonstrated that chitosan hydrogels can boost the antibiofilm effect of incorporated chlorhexidine liposomes for the treatment of chronic wounds.

### 3.4. In Vitro Biocompatibility Assessment

One of the requirements for pharmaceutical formulations is their safety for patients. Therefore, testing the biocompatibility of the formulation is of great importance. The biocompatibility of AZM-liposomal hydrogels was tested in vitro on the HeLa cell line to determine their potential cytotoxicity, which may be contributed by AZM, but also by the ingredients of the formulation. Therefore, in addition to testing the AZM-liposomal hydrogels, empty liposomal hydrogels and empty control-HG (all without AZM) were also tested at concentrations corresponding to the AZM-liposomal hydrogels.

Although the HeLa cell line belongs to cervical cells, it can be used in testing in vitro cytotoxicity/biocompatibility of vaginal formulations [19,36,37].

**Figure 7:**
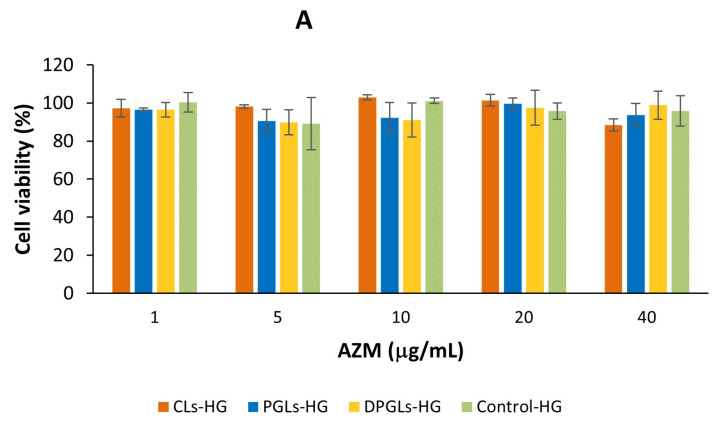

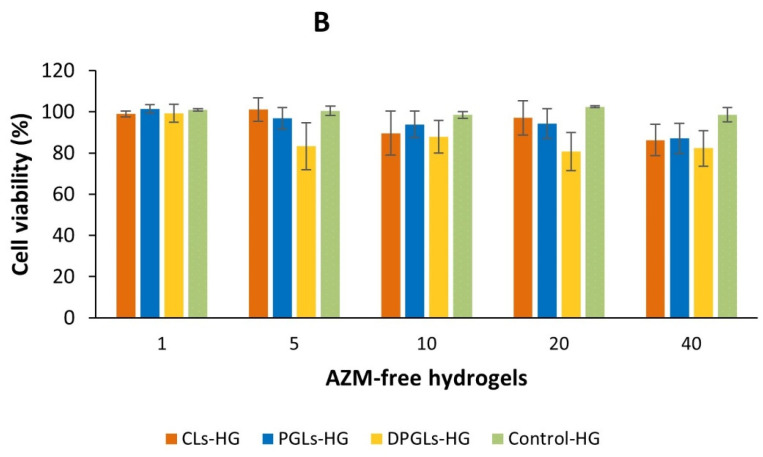
HeLa cell viability after incubation with the different AZM-hydrogels, expressed by AZM concentration (**A**), and with the corresponding AZM-free hydrogels (**B**). The presented values are the mean ± S.D. (*n* = 3).

As shown in Figure 7A, all AZM-liposomal hydrogels were compatible with the HeLa cells. Even at the highest examined concentration (40 µg/mL), which was almost 40-fold higher than the MIC for *S. aureus*, the viability of the cells was greater than 90% (regardless of the type of AZM-liposomes incorporated in chitosan-HG). Testing of the corresponding empty liposomal hydrogels (Figure 7B) confirmed the biocompatibility of all tested liposomal hydrogels. Moreover, the performed studies confirmed the safety of chitosan-HG as a vehicle for the incorporation of AZM-liposomes, where at the highest tested concentration the viability of the cells was higher than 98% (Figure 7). Comparison of the different types of liposomes embedded in chitosan-HG demonstrated that CLs and PGLs were somewhat more tolerable than DPGLs (Figure 7B), although the viability of the cells was greater than 82% at the highest tested concentration. These findings were in agreement with a previous study in which the in vitro cytotoxicity of AZM-liposomes alone was examined and is supposed to be affected also by the negative surface charge of the liposomes [19].

The additional value of the AZM-liposomal hydrogels for the treatment of aerobic vaginitis lies in the immunomodulatory and anti-inflammatory effects of AZM [38,39]. Likewise, chitosan hydrogel made of MMW chitosan has also demonstrated inherent anti-inflammatory effects in vitro [22]. Because of the large amount of water in liposomal hydrogels and its moisturizing effect, in addition to their mucoadhesiveness, which enables enhanced retention of the incorporated drug at mucosal surface, they are suitable for vaginal application and treating various vaginal conditions [7]. Since aerobic vaginitis is characterized by inflammatory changes in the vaginal epithelium, a burning sensation, and epithelial dryness in addition to the excessive growth of aerobic bacteria [2], we assume that AZM-liposomal hydrogels will exhibit multiple therapeutic activities and therefore significantly improve local therapy of aerobic vaginitis. However, these effects should be examined in future studies. Moreover, we also hypothesize that the confirmed inherent anticandidal properties of the chitosan-HG (Table 3) can be beneficial for the prevention of vulvovaginal candidiasis, which might occur as a side-effect of AZM administration.

## 4. Conclusions

This study is the first to report on the potential of AZM-liposomes incorporated in chitosan-HG for the local treatment of aerobic vaginitis. The chitosan-HG improved the antibacterial efficacy of the AZM-liposomes while being physiologically acceptable. By adjusting the physicochemical properties of the AZM-liposomes, prolonged and controlled release of AZM from biocompatible and mucoadhesive AZM-liposomal hydrogels can be achieved, leading to an increased antibacterial effect as well as the desirable mechanical properties relevant for vaginal application. In addition to these features, considering the pathology of aerobic vaginitis (infection and inflammation), the moisturizing effect of AZM-liposomal hydrogels and the accompanying anti-inflammatory abilities of both AZM [38] and chitosan [22], improved local therapy can be achieved.

## Figures and Tables

**Table 1:** Composition of AZM-liposomes.

AZM-Liposomes	EPC (mg)	EPG (mg)	SLPC-80 (mg)	SPC-3 (mg)	PG (g)	AZM (mg)	Buffer, pH 7.5 (g)
CLs	140	10	-	50	-	30	10
PGLs	190	10	-	-	1	30	9
DPGLs	160	10	30	-	1	30	9

AZM, azithromycin; CLs, conventional liposomes with azithromycin; DPGLs, deformable propylene glycol liposomes with azithromycin; EPC, egg phosphatidylcholine; EPG, egg phosphatidylglycerol sodium salt; PG, propylene glycol; PGLs, propylene glycol liposomes with azithromycin; SLPC-80, soybean monoacyl phosphatidylcholine; SPC-3, hydrogenated soybean phosphatidylcholine.

**Table 2:** Physicochemical characteristics of AZM-liposomes.

AZM- Liposomes	Mean Diameter (nm)	PDI	ZP (mV)	Degree of Elasticity	EE (µg AZM/mg Lipid)
CLs	253 ± 3	0.17 ± 0.03	−62.5 ± 0.8	0.5 ± 0.2 *	6.4 ± 0.8
PGLs	216 ± 2	0.19 ± 0.02	−56.2 ± 0.7	13.4 ± 0.5	8.0 ± 0.4
DPGLs	179 ± 1 *	0.20 ± 0.02	−54.3 ± 0.6	16.1 ± 0.7	7.4 ± 0.7

AZM, azithromycin; CLs, conventional liposomes with azithromycin; DPGLs, deformable propylene glycol liposomes with azithromycin; EE, encapsulation efficacy; PDI, polydispersity index; PGLs, propylene glycol liposomes with azithromycin; ZP, zeta potential. Each result represents the mean ± S.D. (*n* = 3). * Significantly different (*p* < 0.05).

**Table 3:** Survival of the test microbes in the chitosan-HG incubated at 37 °C for 28 days, expressed as the logarithmic reduction of the number of microbes after a certain time of incubation.

Test Microbes		*S. aureus* ATCC 6538	*P. aeruginosa* ATCC 9027	*E. coli* ATCC 8739	*C. albicans* ATCC 10231
**Inoculum** **CFU/mL (log_10_)**		3.0 × 10^5^ (5.5)	4.3 × 10^5^ (5.6)	3.5 × 10^5^ (5.5)	4.2 × 10^5^ (5.6)
Log_10_ reduction	2nd day	>4.5	>4.6	>4.5	-
	7th day	>4.5	>4.6	>4.5	-
	14th day	>4.5	>4.6	>4.5	>4.6
	28th day	NI	NI	NI	NI

-: no growth. NI: no increase in the number of viable microbes compared to the previous day. Experiments were performed in duplicate.

**Table 4:** Antibacterial activity of the different AZM-liposomal hydrogels in comparison to corresponding AZM-liposomes.

AZM-Sample	Zone of Inhibition (mm ± S.D.)	
	AZM-Liposomes	AZM-Liposomal Hydrogel
CLs	18.47 ± 0.28	19.07 ± 0.60
PGLs	17.85 ± 0.24	18.50 ± 0.39
DPGLs	17.59 ± 0.22	18.46 ± 0.02
Control *	15.55 ± 0.20	17.05 ± 0.04

* Control denotes free AZM-solution and AZM-hydrogel. The amount of AZM corresponded to the AZM amount in liposomes and liposomal hydrogels. Experiments were performed in duplicate.

## Data Availability

The article contains all the data.

## Abbreviations

AZM, azithromycin; CFUs, colony-forming units; CLs, conventional liposomes with azithromycin; CLs-HG, conventional liposomes with azithromycin incorporated in chitosan hydrogel; control-HG, control hydrogel (azithromycin solution incorporated in chitosan hydrogel); DPGLs, deformable propylene glycol liposomes with azithromycin; DPGLs-HG, deformable propylene glycol liposomes with azithromycin incorporated in chitosan hydrogel; EE, encapsulation efficacy; EPC, egg phosphatidylcholine; EPG, egg phosphatidylglycerol sodium salt; HG, hydrogel; MIC, minimum inhibitory concentration; MMW, medium molecular weight; PDI, polydispersity index; PGLs, propylene glycol liposomes with azithromycin; PGLs-HG, propylene glycol liposomes with azithromycin incorporated in chitosan hydrogel; SLPC-80, monoacyl phosphatidylcholine from soybean; SPC-3, hydrogenated soybean phosphatidylcholine; VFS, vaginal fluid simulant; ZP, zeta potential.
